# PREDICTIVE FACTORS FOR FUNCTIONAL AND MOTOR RECOVERY FOLLOWING SPONTANEOUS INTRACEREBRAL HAEMORRHAGE

**DOI:** 10.2340/jrm.v57.42159

**Published:** 2025-03-06

**Authors:** Shu-Mei YANG, Yen-Heng LIN, Ting-Ju LAI, You-Lin LU, Hsing-Yu CHEN, Hsiao-Ting TSAI, Chueh-Hung WU, Kuo-Chuan WANG, Meng-Ting LIN

**Affiliations:** 1 Department of Physical Medicine and Rehabilitation, National Taiwan University Hospital, College of Medicine, National Taiwan University, Taipei; 2 Department of Physical Medicine and Rehabilitation, National Taiwan University Hospital Hsin-Chu Branch, Hsinchu; 3 Department of Medical Imaging, National Taiwan University Hospital, Taipei; 4 Department of Medical Research, National Taiwan University Hospital Hsin-Chu Branch, Hsin-Chu; 5 Department of Physical Medicine and Rehabilitation, National Taiwan University Hospital Hsin-Chu Branch, Hsinchu; 6 Division of Neurosurgery, Department of Surgery, National Taiwan University Hospital, College of Medicine, National Taiwan University, Taipei, Taiwan

**Keywords:** intracerebral haemorrhage, function, motor recovery, prognostic factors

## Abstract

**Objective:**

Intracerebral haemorrhage significantly impacts patients’ functional and motor recovery. Identifying predictive factors is crucial for enhancing post-intracerebral haemorrhage rehabilitation strategies. This study explores the predictors of functional improvement and motor recovery in intracerebral haemorrhage survivors.

**Design:**

This retrospective cohort study was conducted at a tertiary referral hospital, encompassing patients diagnosed with acute spontaneous intracerebral haemorrhage from 1 June 2019, to 30 June 2023.

**Methods:**

Data on clinical characteristics, activity-based indicators like the initial ability to sit independently without physical assistance and the ability to sit independently for 2 minutes, and haematoma location were analysed to determine their association with functional and motor recovery outcomes, assessed by the modified Rankin Scale, Barthel Index, and Brunnstrom stages.

**Results:**

Among 310 patients, significant predictors for functional outcomes included hypertension, the initial ability to sit independently without physical assistance, the initial ability to sit independently for 2 min, length of hospitalization, and initial National Institute of Health Stroke Scale (NIHSS). For motor recovery, the initial ability to sit independently with-out physical assistance, the initial ability to sit independently for 2 min, 24-h NIHSS, and length of hospitalization were identified as strong predictors for Brunnstrom stage recovery of upper and lower limbs.

**Conclusion:**

Predictive factors including hypertension, early NIHSS, the initial ability to sit independently without physical assistance, the initial ability to sit independently for 2 min, and length of hospitalization play a crucial role in predicting functional and motor recovery after intracerebral haemorrhage.

## LAY ABSTRACT

Intracerebral haemorrhage is a serious type of stroke that can cause significant disability and even death. Understanding the factors that predict recovery in patients with intracerebral haemorrhage can help doctors develop better rehabilitation plans. Our study looked at how well patients regained their ability to perform daily activities and move after intracerebral haemorrhage. We found that certain factors, such as high blood pressure, early neurological evaluations, and activity-based indicators such as the initial ability to sit independently without physical assistance and for 2 min, were important in predicting recovery. These findings highlight the value of early rehabilitation focused on sitting balance and mobility to improve outcomes for intracerebral haemorrhage patients. By identifying these factors, we aim to help healthcare professionals create personalized care strategies, giving patients and families a clearer idea of what to expect during recovery. This knowledge is crucial for optimizing rehabilitation efforts and improving the quality of life for intracerebral haemorrhage survivors.

Intracerebral haemorrhage (ICH) is associated with severe neurological deficit, profound functional impairment, and high mortality, presenting a formidable neurological challenge (1). Spontaneous ICH stands for the rupture of small vessels caused by chronic hypertensive or amyloid angiopathy, accounting for over 80% of ICH patients (2). Precise prediction of functional outcomes and mortality in ICH patients is vital for post-ICH care and rehabilitation programmes. Understanding the likelihood of regaining independence in daily activities provides encouragement for patients and their families, guiding long-term care strategy decisions.

Several prognostic factors were identified to predict mortality and functional deficit for ICH patients. Clinical characteristics such as the Glasgow Coma Scale (GCS) and the National Institutes of Health Stroke Scale (NIHSS) revealed significant influence over the trajectory of functional recovery in ICH patients (3).

Comorbidities and pre-existing health conditions such as hypertension, diabetes, and pre-ICH cognitive impairment revealed the influence on the functional prognosis (3). Radiographic indicators including hematoma location, volume, intraventricular haemorrhage (IVH), and haematoma expansion are crucial for functional independence (4, 5). ICH score was further developed as a useful tool to stratify the severity of ICH for 30-day mortality rates (6). The components of the ICH score include the GCS score, ICH volume, IVH presence, infratentorial ICH, and age; a higher ICH score corresponds to an increased mortality rate (7). Additionally, the ICH score has been validated as a functional prediction tool for up to 12 months in survivors of ICH (7). Our research demonstrated that activity-based indicators, such as the initial ability to sit independently without physical assistance, initial ability to sit independently for 2 min, and earlier, more frequent daily rehabilitation sessions, were significant prognostic factors for patients with stroke undergoing endovascular thrombectomy (8); nevertheless, these factors were rarely investigated in survivors of ICH.

The primary goals of ICH management are to prevent haematoma expansion, reduce cerebral oedema, and manage complications (9). Among commonly used medications, mannitol and corticosteroids have been explored for their potential to reduce cerebral oedema, but evidence supporting their efficacy remains inconsistent (9). Mannitol, an osmotic diuretic, is widely used to lower intracranial pressure, yet multiple studies have found no significant improvement in functional outcomes or mortality (10, 11). Additionally, some findings suggest that mannitol may increase the risk of haematoma expansion, raising concerns over its routine use in acute ICH care (11). Similarly, corticosteroids, particularly dexamethasone, have shown no clear benefits and may be associated with complications such as infections and hyperglycaemia (12, 13). Beyond haematoma control, sedatives and analgesics commonly used in acute ICH care may affect functional recovery (14). Benzodiazepines and opioids, often administered for agitation, anxiety, or pain, can impair motor coordination, balance, and arousal, potentially delaying mobilization and rehabilitation (15, 16). Given these concerns, it is crucial to assess their impact on rehabilitation outcomes, particularly mobility-related functions such as sitting ability and balance.

Most studies have utilized the modified Rankin Scale (mRS) to assess the functional outcome. However, the mRS might not capture specific details regarding individual activities of daily living, and high inter-rater variability was observed due to its broadly defined grading system (17). The Barthel Index (BI), as the indicator of essential daily tasks and useful for rehabilitation monitoring, has proved to be more sensitive than the mRS to predict long-term recovery in ICH patients (18). Furthermore, limited studies have applied the Brunnstrom stages to evaluate motor recovery and predict the likely progress of motor function for the outcomes of ICH survivors, thereby facilitating more effective planning of rehabilitation interventions (19).

Therefore, this study aimed to investigate the predictive factors for functional improvement and motor recovery based on the mRS, BI, and Brunnstrom stages in patients with spontaneous ICH.

## METHODS

### Study setting and population

This cohort study was retrospectively conducted at a tertiary referral hospital and approved by the research ethics committee of the hospital, adhering to the Declaration of Helsinki. The investigation encompassed eligible patients diagnosed consecutively with acute spontaneous ICH documented by brain-computed tomography (CT) scan from 1 June 2019, to 30 June 2023. Those with cerebral aneurysm rupture, arteriovenous malformations, traumatic intracranial haemorrhage, haemorrhagic transformation after ischaemic stroke or brain tumour were excluded (2). The research adhered to the guidelines of Strengthening the Reporting of Observational Studies in Epidemiology (STROBE) (20).

### Collection of predictive factors

From the medical records, we extracted information on basic characteristics such as age, gender, comorbidities, the onset time of ICH, and functional status prior to the ICH. Parameters such as GCS, NIHSS, blood pressure upon arrival at the hospital, laboratory data, and relevant surgery were retrieved retrospectively. Specifics pertaining to the ICH, including IVH, the location, and volume of haematoma were determined from brain CT scans. The location of the haematoma was categorized as caudate, putamen, thalamus, brainstem, cerebellum, or the lobar area, which involved the cortical and/or juxtacortical regions of the cerebral lobes. The haematoma volume was assessed using the initial non-contrast CT scan at the hospital, employing the validated ABC/2 method (21); and the measurement of haematoma was performed by a trained physiatrist with 5-year neurorehabilitation experience. The ICH score assessing GCS, age, ICH volume, IVH, and origin of haemorrhage was calculated accordingly to assess the degree of severity for every participant (7).

Activity-based indicators were utilized to assess functional and motor recovery in patients and were dichotomized to ensure clarity and alignment with the International Classification of Functioning, Disability and Health framework. The first variable, ability to sit independently without physical assistance, was assessed using a modified version of the Functional Independence Measure (FIM). The scale is defined as follows: 0 indicates complete independence; 1 indicates supervision; 2 indicates minimal assistance (the patient exerts 75% or more of their effort); 3 indicates moderate assistance (the patient exerts 50–75% of their effort); 4 indicates maximal assistance (the patient exerts 25–50% of their effort); and 5 indicates total assistance (the patient exerts less than 25% of their effort). Patients were classified as independent if they did not require physical assistance (FIM score 0–1) and dependent if they required physical assistance (FIM score 2–5) (22). The second variable, ability to sit independently for 2 min, was assessed using the “sitting-unsupported” section of the Berg Balance Scale (BBS), which ranges from 0 to 4 (23). The scale is defined as follows: 0 means the patient cannot sit without support for 10 s; 1 means the patient can sit for 10 s; 2 means the patient can sit for 30 s; 3 means the patient can sit for 2 min under supervision; and 4 means the patient can sit safely and securely for 2 min. Patients were categorized as independent if they could sit safely and securely for 2 min (BBS score = 4) and dependent if they could not sit independently for 2 min (BBS score = 0–3). These assessments were conducted during the first physiatrist evaluation, which typically occurred 24–72 h post-stroke.

### Outcome measurements

Functional outcomes were assessed using the mRS scores at 1 month, 3 months, and 6 months post-ICH, along with the BI score at 1 month following the onset of ICH. The mRS is an ordinal scale designed to evaluate functional status semi-quantitatively, with 0 denoting independence and 6 signifying patient mortality. The BI is employed to quantify basic daily activities, ranging from 0 to 100, with scores incremented in intervals of 5. The motor recovery outcome was defined using the Brunnstrom stages, which consist of 6 ordinal stages that describe neurological progress (24). The initial stage signifies total flaccidity of the hemiplegic limbs, while the sixth stage denotes voluntary and individual movements of the assessed limbs without performance interference, indicating superior motor recovery.

### Statistical analysis

Descriptive statistics were conducted with the mean and standard deviation (SD) for continuous variable, median and interquartile range (IQR) for ordinal variables, and percentages for categorical variables. A Shapiro–Wilk test was applied to assess the normality of data distribution. To evaluate the potential impact of a single predictor variable on multicollinearity, the variance inflation factor (VIF) serves as a useful metric, where a VIF value exceeding 10 suggested the presence of multicollinearity. To investigate the correlation between key predictive factors for ICH patients and the ordinal outcomes (mRS and Brunnstrom stages), we initially utilized univariate ordinal logistic regression. Following this, we performed a stepwise multivariate ordinal logistic regression analysis to exclude variables that did not show significance in the cumulative logit model from the univariate analysis. To assess the association between predictors and the continuous outcome (BI), we conducted a generalized linear model (GLM) analysis for univariate factors. Furthermore, multivariate models were developed using PROC GLMSELECT with stepwise selection. Additionally, to examine the relationship between activity-based indicators and the “transfer” item in the BI at 4 weeks, we conducted a univariate logistic regression analysis. The independent variables included initial ability to sit independently without physical assistance and initial ability to sit independently for 2 min, both assessed during the first physiatrist evaluation. The dependent variable was the “transfer” score in the BI at 4 weeks.

Moreover, we analysed the impact of acute-phase medication use within the first 7 days post-admission on post-ICH adverse events and initial activity-based indicators. Medications assessed included mannitol, corticosteroids, benzodiazepines, and opioids. χ^2^ tests were used to examine associations between medication use and post-ICH adverse events. A univariate logistic regression analysis was also performed to evaluate the association between these medications and activity-based indicators, including both the ability to sit independently without physical assistance and the ability to sit independently for 2 min. To determine whether surgery influenced hospitalization duration, we conducted an independent *t*-test comparing hospital stay lengths between surgical and non-surgical groups. In addition, univariate logistic regression was performed to assess the association between hospitalization length and Brunnstrom stage at 4 and 12 weeks in both groups.

A two-sided α value below 0.05 indicated statistical significance. All statistical evaluations were executed using SAS software (Version 9.4, SAS Institute Inc, Cary, NC, USA).

## RESULTS

The clinical and rehabilitation characteristics, including brain imaging, of the patient cohort are presented in Table I. A total of 310 patients were eligible for the analysis, with 183 (59.03%) being male and a mean age of 63.44±13.33 years. Hypertension (71.52%), dyslipidaemia (26.77%), and diabetes mellitus (24.52%) were the most common risk factors (Table I). The majority of patients (94.19%) had functional independence before the stroke event, as indicated by their mRS scores of 0–2. During hospitalization, 48.84% of patients experienced immobility-related complications. Among these, pneumonia (34.8%), pressure injuries (28.7%), and urinary tract infections (19.4%) were the most common adverse events.

**Table I:** Baseline characteristics of patients

Parameter	Value
Number of patients	310
Baseline status	
Age (SD), year	63.44 ± 13.33
Male sex, *n* (%)	183 (59.03%)
Body mass index (SD), kg/m^2^	24.58 ± 4.50
Premorbid mRS 0–2, *n* (%)	292 (94.19%)
Risk factors, *n* (%)	
Hypertension	221 (71.52%)
Diabetes mellitus	76 (24.52%)
Dyslipidaemia	83 (26.77%)
Atrial fibrillation	15 (4.84%)
Peripheral arterial occlusion disease	2 (0.65%)
Ischemic heart disease	25 (8.06%)
Previous history of ischaemic stroke	33 (10.65%)
Previous history of haemorrhagic stroke	37 (11.94%)
Smoking	44 (14.24%)
Initial laboratory data, mean (SD)	
Hb (SD), g/dL	13.88 ± 2.21
Platelet count (SD), 10^3^/uL	227.46 ± 71.76
WBC count (SD), 10^3^/uL	9.20 ± 3.27
Blood glucose (SD), mg/dL	149.04 ± 60.06
CRP (SD), mg/dL	2.55 ± 5.33
Albumin (SD), g/dL	3.79 ± 0.49
Total cholesterol (SD), mg/dL	177.55 ± 43.78
eGFR (SD), mL/minute/m^2^	87.20 ± 38.10
Previous medication use	
Antiplatelet drugs	22 (7.12%)
Anticoagulant drugs	35 (11.29%)
Antihypertensive drugs	111 (35.81%)
Lipid-lowering drugs	50 (16.18%)
ICH-related characteristics	
Initial GCS score (IQR)	14 (5)
Initial NIHSS (IQR)	13 (10)
NIHSS 24 h after ER arrival (IQR)	12 (10)
SBP on ER arrival (SD), mmHg	178.5 ± 32.94
SBP 24 h after ER arrival (SD), mmHg	136.45 ± 17.19
SBP on first rehabilitation (SD), mmHg	134.46 ± 16.04
Surgical management, *n* (%)	78 (25.16%)
Haematoma location, *n* (%)	
Lobar	73 (23.55%)
Putamen	89 (28.71%)
Caudate nucleus	3 (0.97%)
Thalamus	96 (30.97%)
Brainstem	20 (6.45%)
Cerebellum	20 (6.45%)
ICH volume (SD), ml	26.24 ± 26.05
Intraventricular haemorrhage, *n* (%)	101 (32.5 8%)
ICH score (IQR)	1 (2)
Medication use post-ICH during acute care	
Mannitol	79 (25.5%)
Corticosteroids	52 (16.8%)
Benzodiazepines	148 (47.7%)
Opioids	56 (18.1%)
Activity-based indicators	
Onset-to-first PT time (SD), days	10.13 (21.91)
Initial ability to sit independently without physical assistance, *n* (%)	12 (3.92%)
Initial ability to sit independently for 2 min, *n* (%)	73 (23.85)
Immobility adverse events, *n* (%)	148 (48.84%)
Pressure injury	89 (28.7%)
Deep vein thrombosis	6 (1.9%)
Pneumonia	108 (34.8%)
Urinary tract infection	60 (19.4%)
Total hospitalization days (SD)	41.62 (23.53%)
mRS at 4 weeks (IQR)	4 (0)
mRS at 12 weeks (IQR)	4 (1)
BS of proximal UL at 4 weeks (IQR)	4 (2)
BS of distal UL at 4 weeks (IQR)	4 (3)
BS of LL at 4 weeks (IQR)	4 (2)
BS of proximal UL at 12 weeks (IQR)	5 (3)
BS of distal UL at 12 weeks (IQR)	5 (2.5)
BS of LL at 12 weeks (IQR)	5 (2)
Barthel Index at 4 weeks (IQR)	25 (40)

mRS: modified Rankin Scale; Hb: haemoglobin; WBC: white blood cell; CRP: C-reactive protein; eGFR: estimated glomerular filtration rate; GCS: Glasgow Coma Scale; NIHSS: National Institute of Health Stroke Scale; SBP: systolic blood pressure; DBP: diastolic blood pressure; ER: emergency room; PT: physical therapy; ICH: intracerebral haemorrhage; BS: Brunnstrom stage; UL: upper limb; LL: lower limb.

### Correlations between predictive variables and functional status

*Predictive factors associated with mRS at 4 weeks and 12 weeks.* Initial univariate analysis identified a spectrum of predictors linked to less favourable functional outcomes at 4 weeks, encompassing hypertension, CRP elevation, lower GCS scores at presentation, increased baseline NIHSS and NIHSS 24 h after ER arrival, as well as higher SBP within 24 h post-ER admission. Other notable factors were complications related to immobility, prolonged hospital stays, diminished initial ability to sit independently without physical assistance, compromised ability to sit independently for 2 min, the occurrence of IVH, and elevated ICH scores (Table SIA). Subsequent multivariate analysis (Table II) showed hypertension, impaired ability to sit independently without physical assistance, and prolonged hospitalization as significant predictors of poor functional outcomes at 4 weeks.

**Table II:** Multivariate stepwise analysis: Factors associated with mRS at 4 weeks and 12 weeks

Parameters	mRS at 4 weeks	
	Adjusted OR (95% CI)	*p*-value
Hypertension	3.369 (1.191–9.526)	0.022*
Initial ability to sit independently without physical assistance	0.534 (0.368–0.776)	0.001**
Total hospitalization days	1.047 (1.019–1.075)	0.001**

Parameters	mRS at 12 weeks	

	Adjusted OR (95% CI)	*p*-value

Initial ability to sit independently for 2 min	0.255 (0.130–0.502)	<0.0001**

mRS: modified Rankin Scale; OR: odds ratio; CI: confidence interval.

* *p* < 0.05;

** *p* < 0.01.

At 12 weeks, predictors of adverse functional outcomes encompassed older age, hypertension, atrial fibrillation, reduced albumin level, usage of lipid-lowering medications, lower initial GCS scores, elevated NIHSS scores at onset and 24 h post-ER admission, presence of immobility adverse events, extended hospital stays, longer onset-to-first PT time, reduced ability to sit independently without physical assistance, impaired ability to sit independently for 2 min, putamen haematoma, increased ICH volume and ICH score, and the presence of IVH (Table S1B). The stepwise multivariate analysis (Table II) identified compromised ability to sit independently for 2 min as a critical indicator of 12-week functional prognosis.

*Predictive factors associated with BI at 4 weeks.* The correlation between the BI at 4 weeks and a broad spectrum of clinical, laboratory, and imaging variables was examined through linear regression analysis, as detailed in Table III. Univariate analysis highlighted several factors negatively affecting the BI: advanced age, hypertension, elevated serum glucose and CRP levels, decreased serum albumin and eGFR, lower initial GCS scores, higher NIHSS scores both initially and 24 h post-ER, immobility-related complications, extended hospitalizations, impaired ability to sit independently without physical assistance, compromised ability to sit independently for 2 min, thalamic haematoma locations, larger ICH volumes, the presence of intraventricular haemorrhage, and higher ICH scores. The multivariate analysis further identified age, hypertension, initial NIHSS score, and initial ability to sit independently for 2 min as key factors significantly correlated with the BI at 4 weeks, each demonstrating a statistically significant association (*p* < 0.05).

**Table III:** Predictive factors associated with Barthel Index at 4 weeks

Parameters	Univariate analysis		Multivariate analysis	
	β coefficient	*p*-value	β coefficient	*p*-value
Age, years	–0.258 (–0.503, –0.012)	0.0399*	–0.440 (–0.717, –0.187)	0.0016**
Hypertension	–13.229 (–20.326, –6.131)	0.0003**	–8.585 (–16.524, –0.990)	0.0348*
Blood glucose, mg/dL	–0.074 (–0.131, –0.017)	0.0116*		
CRP, mg/dL	–0.775 (–1.346, –0.204)	0.008**		
Albumin, g/dL	7.044 (0.230, 13.859)	0.0428*		
eGFR, mL/min/m^2^	0.095 (0.001, 0.189)	0.0468*		
Initial GCS score	2.004 (1.051, 2.957)	<0.0001**		
Initial NIHSS	–1.304 (–1.821, –0.787)	<0.0001**	–1.077 (–1.781, –0.593)	0.0006**
NIHSS 24 h after ER arrival	–1.816 (–2.380, –1.251)	<0.0001**		
Hematoma location, thalamus	–7.110 (–13.972, –0.248)	0.0423*		
ICH volume, ml	–0.205 (–0.333, –0.077)	0.0018**		
Intraventricular haemorrhage	–10.483 (–17.257, –3.710)	0.0026**		
ICH score	–8.314 (–11.353, –5.275)	<0.0001**		
Immobility adverse events	–12.2 (–18.707, –5.693)	0.0003**		
Total hospitalization days	–0.365 (–0.513, –0.218)	<0.0001**		
Initial ability to sit independently without physical assistance	12.233 (9.814, 14.652)	<0.0001**		
Initial ability to sit independently for 2 min	12.544 (10.281, 14.807)	<0.0001**	6.888 (2.757,10.215)	0.0005

CRP: C-reactive protein; eGFR: estimated glomerular filtration rate; GCS: Glasgow Coma Scale; DBP: diastolic blood pressure; ICH: intracerebral haemorrhage; CI: confidence interval.

* *p* < 0.05;

** *p* < 0.01.

The analysis of the association between initial activity-based indicators and the “transfer” item in the BI at 4 weeks showed that patients who were unable to sit independently without physical assistance had significantly higher odds of poor transfer ability (odds ratio [OR] 2.046, *p* = 0.0297). Similarly, patients who were unable to sit independently for 2 min had significantly higher odds of poor transfer function (OR 4.016, *p* < 0.0001) (Table SII).

### Correlations between predictive variables and motor recovery

*Predictive factors associated with upper limb recovery.* Analysis at 4 weeks identified factors such as age, absence of prior ischaemic stroke, higher platelet counts, lower initial GCS scores, increased NIHSS scores at onset and 24 h post-ER, surgical interventions, complications from immobility, prolonged hospital stays, increased ICH volumes, elevated ICH scores, and compromised sitting capabilities affecting upper limb recovery. Notably, putamen haematoma negatively influenced recovery, whereas cerebellar location showed a positive association (Table SIIIA and B). Multivariate analysis underscored the importance of initial ability to sit independently for 2 min and NIHSS scores 24 h post-ER as predictors of motor recovery outcomes for both proximal and distal upper limbs at 4 weeks (Table IV).

**Table IV:** Multivariate stepwise analysis: factors associated with Brunnstrom stage of hemiplegic limb at 4 weeks and 12 weeks

Parameters	Upper proximal limb at 4 weeks		Parameters	Upper proximal limb at 12 weeks	
	Adjusted OR (95% CI)	*p*-value		Adjusted OR (95% CI)	*p*-value
Initial ability to sit independently for 2 min	0.674 (0.562–0.807)	<0.0001**	Initial ability to sit independently without physical assistance	0.599 (0.435–0.825)	0.0017**
NIHSS 24 h after ER arrival	1.065 (1.035–1.096)	<0.0001**	NIHSS 24 h after ER arrival	1.069 (1.018–1.121)	0.0068**

Parameters	Upper distal limb at 4 weeks		Parameters	Upper distal limb at 12 weeks	
	Adjusted OR (95% CI)	*p*-value		Adjusted OR (95% CI)	*p*-value

Initial ability to sit independently for 2 min	0.757 (0.651–0.879)	0.0003**	Initial ability to sit independently without physical assistance	0.592 (0.438–0.802)	0.0007**
NIHSS 24 h after ER arrival	1.069 (1.040–1.098)	<0.0001**	Hospitalization days	1.016 (1.004–1.029)	0.011*

Parameters	Lower limb at 4 weeks		Parameters	Lower limb at 12 weeks	
	Adjusted OR (95% CI)	*p*-value		Adjusted OR (95% CI)	*p*-value

Initial ability to sit independently for 2 min	0.61 (0.490–0.756)	<0.0001**	Initial ability to sit independently without physical assistance	0.553 (0.381–0.803)	0.0018**
NIHSS 24 h after ER arrival	1.083 (1.046–1.122)	<0.0001**	NIHSS 24 h after ER arrival	1.084 (1.018–1.153)	0.0114*

NIHSS: National Institute of Health Stroke Scale; ER: emergency room; OR: odds ratio; CI: confidence interval.

* *p* < 0.05;

** *p* < 0.01.

At 12 weeks, the analysis reiterated the influence of similar factors (Table SIVA and B), with ability to sit independently without physical assistance and NIHSS scores 24 h after ER admission emerging as key predictors for upper limb recovery (Table IV).

*Predictive factors associated with lower limb recovery.* For lower limb recovery at 4 weeks, age, absence of previous history of ischaemic stroke, GCS scores, baseline and post-24-h ER NIHSS, surgical management, increased ICH volume and ICH score, and presence of IVH, with immobility complications and extended hospital stays further complicating recovery. Notably, cerebellar haematoma locations were again associated with more favourable outcomes (Table SVA). The 12-week analysis showed that lower initial GCS scores, elevated baseline and post-24-h ER NIHSS, extended hospital stays, impaired ability to sit independently without physical assistance and to sit independently for 2 min, increased ICH volume and ICH score were linked to poorer outcomes, while cerebellar location showed an association with better outcome (Table SVB).

In the multivariate context (Table IV), impaired ability to sit independently for 2 min and NIHSS 24 h post-ER were significant for poor lower limb recovery at 4 weeks, while initial ability to sit independently without physical assistance and NIHSS 24 h post-ER were significant predictors at 12 weeks.

### Association between medication use during acute care post-ICH, post-ICH immobility-related adverse events, and activity-based indicators

Among the medications analysed, corticosteroid use and benzodiazepine use were significantly associated with a higher incidence of adverse events (Table SVIA). In contrast, neither mannitol nor opioid use showed a significant association with post-ICH complications.

For activity-based indicators, corticosteroid use was significantly associated with a reduced ability to sit independently for 2 min (Table SVIB). However, no significant effects on sitting ability were observed with benzodiazepines, mannitol, or opioids.

### Association between surgical status, hospitalization length, and motor recovery

Surgical patients had a slightly longer hospital stay (45.40 ± 23.20 days) compared with non-surgical patients (40.35 ± 23.56 days); however, this difference was not statistically significant (*p* = 0.1029) (Table SVIIA). Among non-surgical patients, a longer hospital stay was significantly associated with poorer motor recovery in both the upper and lower limbs at 4 and 12 weeks (*p* < 0.01). In contrast, hospitalization duration in the surgical group was not significantly correlated with motor recovery, except for an association with lower limb Brunnstrom stage at 12 weeks (*p* = 0.0249) (Table SVIIB).

## DISCUSSION

Our study comprehensively evaluated the predictive factors for functional and motor recovery in patients following ICH, highlighting the significant role of clinical and activity-based indicators in determining patient outcomes. We found that hypertension, initial ability to sit independently without physical assistance, length of hospitalization, initial ability to sit independently for 2 min, and the initial NIHSS scores were critical in predicting functional outcomes, as measured by the mRS and BI. Moreover, our analysis revealed that initial ability to sit independently for 2 min, 24-h NIHSS and length of hospitalization were robust predictors of motor recovery in both upper and lower limbs. The predictive importance of variables including hypertension, initial NIHSS scores, initial ability to sit independently without physical assistance and to sit independently for 2 min in determining functional recovery aligns with prior research findings (19, 25–27).

The prognostic value of factors of initial NIHSS scores in predicting functional outcomes corroborates with existing literature (26, 27). The NIHSS, a straightforward and validated clinical tool, proves valuable for numerous reasons, particularly its ability to track clinical status. It is designed to highlight clinical fluctuations and the immediate impacts of therapeutic interventions. Originally developed for ischaemic stroke, its utility has extended to ICH monitoring, reflecting its adaptability (28, 29). Given that ICH is characterized by its dynamic nature, with potential for neurological decline within the initial 24 to 48 h due to factors like oedema, decreased perilesional blood flow, and haematoma growth, our study accentuates the critical role of early neurological evaluation using the NIHSS score (30, 31). Notably, our analysis identified NIHSS scores, especially those recorded 24 h after emergency room admission, as strong indicators of motor function recovery in both upper and lower extremities. Considering that approximately 17% of ICH sufferers encounter early (within 24 h) or subsequent (1 to 7 days) neurological worsening due to various complications such as haemorrhage expansion, hydrocephalus, or surrounding oedema, a delayed approach to prognosis could offer more accurate predictions, highlighting the pivotal role of timely and ongoing neurological assessments in ICH management (32).

Our research expands on previous findings, highlighting the critical role of the capacity for sitting up and maintaining balance in post-stroke functional recovery (19, 33–35). These abilities are foundational for daily living tasks such as personal hygiene and feeding, which demand more than mere upper limb function; they require the coordinated effort of trunk and lower limb muscles to counteract gravity (34). The complexity of sitting up involves not just physical strength but also the integration of sensory and motor skills, often compromised by stroke-induced impairments like cognitive dysfunction and spasticity (35). Addressing these deficits through targeted physical therapy, particularly exercises aimed at strengthening the trunk and limb muscles, is pivotal for rehabilitation. The essence of sitting balance extends beyond static stability; it is integral for engaging in activities of ADLs and is the basis for more complex movements needed for transfers and mobility (35). Our findings demonstrate a significant association between initial sitting ability and transfer ability at 4 weeks post-ICH. Patients who required physical assistance to sit independently had twice the odds of poor transfer outcomes, while those unable to maintain an independent sitting position for 2 min faced an almost fourfold increased risk of impaired transfers. Prior studies have shown that postural stability is fundamental for weight shifting, balance adjustments, and safe transfers, which are key components of the BI transfer item (36). These findings underscore the necessity of early postural control training to enhance transfer performance and overall functional recovery.

The interdependence of cognitive, motor, and sensory functions in maintaining balance underscores the necessity for comprehensive assessments and tailored rehabilitation strategies early in the stroke recovery process (23). While early rehabilitation programmes often emphasize activity frequency and general mobility (37), our findings advocate for the importance of addressing core motor functions and balance-specific strategies early in the recovery process. This targeted approach not only aids immediate recovery but also establishes the foundation for complex functional activities like transfers and independent mobility. By integrating assessments and interventions tailored to these specific needs, rehabilitation can better align with the unique recovery pathways of post-stroke patients, maximizing their potential for sustained improvements (38).

Early rehabilitation offers significant benefits for patients with ischaemic and haemorrhagic strokes, including improved motor recovery, greater functional independence, and a reduced risk of complications such as deep vein thrombosis and pneumonia (39–41). Initiating therapy within 24–72 h post-stroke takes advantage of the acute phase of heightened neuroplasticity, fostering faster recovery and better long-term outcomes (39). In Taiwan, ICH patients typically receive physiatrist consultations within 24–72 h of admission for bedside evaluations and therapy prescriptions. However, therapy initiation may be delayed by 1–2 days due to administrative processes or holidays. If patients are transferred from the intensive care unit (ICU) to general wards before starting therapy, additional delays may occur as therapy requests must be reinitiated. In addition, Taiwan’s National Health Insurance may require physician pre-authorization for rehabilitation services, rather than allowing direct access through therapists, which can result in administrative delays. Moreover, unstable clinical or neurological conditions can further postpone rehabilitation. These systemic factors contributed to the observed average of 10 days to the first PT session in our study. To improve care for ICH patients, addressing delays in rehabilitation initiation is critical. Streamlining administrative processes and enhancing communication among neurologists, neurosurgeons, physiatrists, and rehabilitation therapists can significantly reduce the time to therapy. Establishing standardized protocols for early rehabilitation with clearly defined roles and responsibilities ensures seamless transitions of care, particularly between ICU and general wards. A multidisciplinary approach is vital for recovery, integrating neurological expertise with personalized rehabilitation strategies. Neurologists and neurosurgeons provide critical insights into acute stability, physiatrists assess functional abilities and design tailored therapy plans, and therapists execute these plans to improve mobility and independence (42, 43). Timely, coordinated interventions optimize the neuroplasticity window, reduce immobility-related complications, and enhance recovery. This collaborative model can ultimately improve patient outcomes, quality of life, and healthcare resource utilization.

The prognostic utility of haematoma volume in ICH is well established, serving as a crucial and straightforward indicator for patient outcomes (44–46). The detrimental effects of ICH unfold in 2 distinct stages: an initial mechanical disruption where bleeding into brain structures causes tissue compression and displacement, followed by a secondary phase characterized by complications such as haematoma growth or surrounding oedema (47). Furthermore, a clear association exists between haemorrhage volume and the initial assessment scores on the NIHSS, highlighting its role in gauging the severity of ICH (28). Although haematoma volume has been consistently recognized as a critical factor in various studies and our preliminary analyses, its influence appeared to be less significant in our more comprehensive multivariate analysis. This discrepancy might stem from the variability in haematoma sizes depending on their location within the brain. Recent research in 2023 underscores the variability in ICH outcomes based on the precise location of the haematoma, suggesting that certain volume thresholds specific to various brain regions are more indicative of adverse outcomes. For example, exceeding 48 mL in lobar regions or 41 mL in the putamen and external capsule markedly increases the risk of unfavourable outcomes, with distinct thresholds identified for other areas such as the internal capsule, thalamus, cerebellum, and brainstem (48). Given these insights, it becomes clear that when evaluating ICH patients the location-specific size of the haematoma should be a key consideration, especially as our study encompasses a wide range of brain locations, including the lobar areas, deep nuclei, thalamus, brainstem, and cerebellum. This tailored approach to assessing haematoma volume could refine prognosis accuracy and inform more personalized treatment strategies for ICH patients.

In our study, univariate analysis revealed a trend suggesting that the location of the haematoma significantly influences patient outcomes. Haemorrhages in the intraventricular and putamen regions were linked to less favourable recoveries, whereas those in the cerebellar region were associated with more positive outcomes. The relationship between IVH and negative outcomes in ICH cases is well documented, largely attributed to the pathophysiological effects of IVH (49). The blockage of cerebrospinal fluid pathways by blood and its derivatives, resulting in obstructive hydrocephalus and meningeal irritation, is a critical factor, even though it may not always cause an increase in intracranial pressure (50). However, the link between IVH and severe disabilities appeared less significant in our multivariate analysis, possibly due to the overshadowing effect of direct damage from the primary intraparenchymal haemorrhage to the neuronal tissue. Moreover, the diminished correlation between IVH and primary outcomes or mortality could also stem from differences in treatment approaches, as previous research suggests that specific interventions, notably the insertion of a ventricular drain, might offer advantages (51). Comparative studies indicate that individuals with deep grey matter haemorrhages experience a more significant decline in health-related quality of life 3 months after the event compared with those with lobar haemorrhages (52). This unique correlation with deep ICH locations might stem from the profound motor impairments and widespread cortical dysfunction, such as issues with language, sensation, and spatial awareness, typically caused by haemorrhages in the basal ganglia. Alternatively, this disparity could result from the different causes of lobar haemorrhages, frequently associated with amyloid angiopathy, and deep haemorrhages, typically due to hypertensive small vessel disease, leading to more extensive local tissue damage (52). Data from the INTERACT trials on infratentorial ICH show that patients with brainstem ICH face a higher mortality risk than those with cerebellar ICH, although there is no significant difference in functional outcomes (53). Furthermore, a study in 2020 revealed that cerebellar ICH patients often have milder initial symptoms and achieve better functional recoveries compared with those with brainstem ICH. These findings, together with our research, highlight the importance of further exploration into the effects of haematoma location on ICH outcomes (54).

A unique aspect of our study is the application of the Brunnstrom stages to evaluate motor recovery, which has been less commonly used in ICH research. By incorporating this approach, our study offers a nuanced understanding of motor recovery progress, enabling more tailored and potentially effective rehabilitation plans. This complements existing literature by providing a more detailed analysis of motor function recovery stages in ICH patients, which can guide the timing and intensity of therapeutic interventions (55).

Our findings suggest that medications commonly used in acute ICH management may influence early functional recovery, particularly in relation to immobility-related complications and sitting ability. Corticosteroids and benzodiazepines were associated with higher rates of immobility-related complications, while corticosteroids were linked to impaired sitting balance. These findings align with previous research indicating that corticosteroids may contribute to muscle weakness, metabolic disturbances, and an increased risk of infections, all of which could delay early mobilization efforts (9, 12). Similarly, benzodiazepines are known to impair motor coordination, postural stability, and cognitive function (56), which may contribute to immobility-related complications in ICH patients. In contrast, mannitol and opioids did not show significant associations with either immobility-related complications or sitting ability in our study. Although mannitol is frequently used to manage cerebral oedema, its role in functional recovery remains uncertain, as prior studies have failed to demonstrate clear benefits (9–11). Opioids, commonly prescribed for pain control, may lead to sedation and dizziness (57), but their direct impact on mobility-related outcomes requires further investigation. Given these findings, medication use in ICH patients should be carefully considered to minimize potential negative effects on rehabilitation. Optimizing medication strategies to balance symptom control with functional recovery should be a priority in ICH rehabilitation protocols.

Our study revealed a significant association between hospitalization length and motor recovery in non-surgical ICH patients, whereas this relationship is weaker in those who underwent surgery. Several factors may explain this difference. In non-surgical patients, hospitalization length may be largely influenced by the severity of neurological deficits, functional impairments, and complications related to immobility (58). As these patients depend on rehabilitation for recovery, a longer hospital stay could reflect greater initial impairment and slower motor recovery, as seen in the stronger correlation with Brunnstrom stages at 4 and 12 weeks. In contrast, surgical patients may experience a different recovery trajectory. Haematoma evacuation or decompression may relieve pressure on surrounding brain structures, potentially allowing faster neurological improvement (59, 60). However, hospitalization length in this group is more affected by post-surgical monitoring, ICU stay, and surgical complications rather than motor recovery alone (61), which could explain the weaker correlation with Brunnstrom stages. Additionally, selection bias may play a role, as surgical patients often meet specific clinical criteria that may influence both hospitalization length and recovery outcomes.

### Limitations

Our study has several limitations that warrant consideration. First, its retrospective design inherently constrains our ability to establish causality between predictive factors and patient outcomes. As with all retrospective analyses, there is a risk of unmeasured confounders that could potentially impact the results. Although the Brunnstrom stages offer a detailed assessment of motor recovery, their subjective nature and the potential for inter-rater variability may compromise the reliability of our findings. Furthermore, our results, obtained from a single-centre cohort, may not fully reflect the diversity of the broader ICH patient population, potentially affecting the external validity of our conclusions. The recruitment of patients from a tertiary referral hospital might introduce referral bias, as these individuals could have more complex conditions or complications compared with the general ICH population. Another limitation is the lack of direct cognitive function assessments, which may influence the ability to achieve and maintain a sitting position. While we used the GCS as a surrogate measure of consciousness, we did not evaluate specific cognitive domains such as attention, executive function, or spatial awareness, all of which affect postural control and balance. Given the association between cognitive impairment, poor motor recovery, and immobility-related complications, future studies should include comprehensive cognitive assessments. Lastly, our study did not consider all potential confounding factors, such as the specifics of rehabilitation interventions and the intensity of therapy, which could significantly influence recovery outcomes. Prospective, multicentre studies with standardized data collection protocols are needed to confirm and expand upon our findings.

### Conclusions

Clinical parameters such as hypertension and early NIHSS scores, combined with activity-based indicators like initial ability to sit independently without physical assistance, initial ability to sit independently for 2 min, and length of hospitalization, are crucial for predicting functional and motor recovery after ICH. Future research should examine the influence of cognitive function on activity-based indicators and the impact of acute-phase medications on functional recovery.

## Supplementary Material

PREDICTIVE FACTORS FOR FUNCTIONAL AND MOTOR RECOVERY FOLLOWING SPONTANEOUS INTRACEREBRAL HAEMORRHAGE
