# Supplementary material for: PREDICTIVE FACTORS FOR FUNCTIONAL AND MOTOR RECOVERY FOLLOWING SPONTANEOUS INTRACEREBRAL HAEMORRHAGE
Source: J Rehabil Med. 2025 Mar 6;57:42159. doi: 10.2340/jrm.v57.42159 (PMC11898305; doi:10.2340/jrm.v57.42159)
Supplement: PREDICTIVE FACTORS FOR FUNCTIONAL AND MOTOR RECOVERY FOLLOWING SPONTANEOUS INTRACEREBRAL HAEMORRHAGE [file JRM-57-42159-s1.pdf]

Supplementary material has been published as submitted. It has not been copyedited, or typeset by Journal of Rehabilitation Medicine

S-Table 1a Univariate analysis: Factors associated with mRS at 4 weeks

Parameter	Unadjusted OR (95% CI)	p value
**Baseline status**		
Age	1.01 (0.988-1.032)	0.3748
Male	0.775 (0.428-1.404)	0.4009
BMI	1.027 (0.96-1.098)	0.4382
Premorbid mRS	1.135 (0.799-1.611)	0.4794
**Risk factors**		
Hypertension	2.684 (1.4-5.146)	0.003**
Diabetes mellitus	1.043 (0.527-2.068)	0.9032
Dyslipidemia	1.005 (0.52-1.945)	0.9876
Atrial fibrillation	2.237 (0.511-9.786)	0.2851
Peripheral Arterial Occlusion Disease	2.119 (0.049-90.777)	0.6953
Ischemic heart disease	0.865 (0.312-2.395)	0.7799
Previous history of ischemic stroke	1.32 (0.474-3.677)	0.5954
Previous history of hemorrhagic stroke	0.895 (0.368-2.177)	0.8075
Smoking	0.655 (0.307-1.394)	0.2717
**Initial laboratory data**		
Hb	0.91 (0.795-1.041)	0.1679
Platelet count	1.022 (0.998-1.006)	0.414
WBC count	1.025 (0.936-1.122)	0.5973
Blood glucose	1.002 (0.997-1.008)	0.42
CRP	1.16 (1.015-1.327)	0.0293*
Albumin	0.645 (0.301-1.383)	0.2601
Total cholesterol	0.999 (0.991-1.006)	0.73
eGFR	0.996 (0.989-1.004)	0.3499
**Previous medication use**		

Antiplatelet drugs	1.033 (0.325-3.283)	0.9566
Anticoagulant drugs	0.832 (0.337-2.058)	0.6909
Antihypertensive drugs	0.885 (0.484-1.62)	0.6928
Lipid-lowering drugs	1.177 (0.522-2.653)	0.6938
**ICH-related characteristics**		
Initial GCS score	0.871 (0.789-0.962)	0.0067**
Initial NIHSS	1.107 (1.049-1.167)	0.0002**
NIHSS 24 hours after ER arrival	1.129 (1.047-1.217)	0.0016**
SBP on ER arrival	1.006 (0.997-1.015)	0.1988
SBP 24 hours after ER arrival	1.024 (1.008-1.041)	0.0031**
SBP on first rehabilitation	1.009 (0.991-1.028)	0.3156
Surgical management	1.845 (0.856-3.977)	0.1181
Hematoma location		
Lobar	0.994 (0.498-1.985)	0.9869
Putamen	0.704 (0.376-1.317)	0.2721
Caudate nucleus	2.127 (0.097-46.519)	0.6315
Thalamus	1.87 (0.911-3.84)	0.088
Brainstem	0.719 (0.248-2.082)	0.5433
Cerebellum	0.719 (0.248-2.082)	0.5433
ICH volume	1.012 (0.999-1.025)	0.066
Intraventricular hemorrhage	3.127 (1.337-7.312)	0.0085**
ICH score (IQR)	1.836 (1.287-2.619)	0.0008**
**Activity-based indicators**		
Onset-to-first PT time	1.01 (0.999-1.022)	0.0828
Initial ability to sit independently without physical assistance	0.567 (0.453-0.71)	<0.0001**
Initial ability to sit independently for 2 minutes	0.527 (0.406-0.683)	<0.0001**
Immobility adverse events	2.345 (1.191-4.616)	0.0137*
Total hospitalization days	1.025 (1.011-1.039)	0.0004**

Abbreviation: mRS, modified Rankin Scale; BMI, body mass index; Hb, hemoglobin; WBC, white blood cell; CRP, C-reactive protein; eGFR, estimated glomerular filtration rate; GCS, Glasgow Coma Scale; NIHSS, National Institute of Health Stroke Scale; SBP, systolic blood pressure; DBP, diastolic blood pressure; ER: emergency room; PT, physical therapy; ICH, intracerebral hemorrhage; OR, odds ratio; CI, confidence interval. * p < 0.05; **p < 0.01

S-Table 1b Univariate analysis: Factors associated with mRS at 12 weeks

Parameter	Unadjusted OR (95% CI)	p value
**Baseline status**		
Age	1.039 (1.016-1.062)	0.0006**
Male	0.941 (0.558-1.587)	0.8186
BMI	1.003 (0.947-1.063)	0.9133
Premorbid mRS	1.319 (0.936-1.859)	0.1141
**Risk factors**		
Hypertension	2.063 (1.152-3.694)	0.0149*
Diabetes mellitus	1.669 (0.885-3.146)	0.1136
Dyslipidemia	1.416 (0.787-2.548)	0.2453
Atrial fibrillation	5.86 (1.328-25.87)	0.0196*
Peripheral Arterial Occlusion Disease	3.099 (0.356-27.006)	0.3058
Ischemic heart disease	0.658 (0.275-1.575)	0.347
Previous history of ischemic stroke	1.801 (0.75-4.324)	0.1877
Previous history of hemorrhagic stroke	2.404 (0.991-5.835)	0.0525
Smoking	0.848 (0.388-1.854)	0.6795
**Initial laboratory data**		
Hb	0.915 (0.802-1.043)	0.1834
Platelet count	1 (0.996-1.003)	0.8644
WBC count	0.981 (0.905-1.064)	0.6441
Blood glucose	1.004 (0.999-1.009)	0.1002
CRP	1.062 (0.988-1.142)	0.1048
Albumin	0.558 (0.312-0.998)	0.0493*
Total cholesterol	0.993 (0.985-1)	0.0596

eGFR	0.997 (0.99-1.004)	0.3586
**Previous medication use**		
Antiplatelet drugs	1.869 (0.606-5.765)	0.2765
Anticoagulant drugs	0.923 (0.413-2.065)	0.8463
Antihypertensive drugs	1.577 (0.904-2.75)	0.1087
Lipid-lowering drugs	2.153 (1.027-4.515)	0.0424*
**ICH-related characteristics**		
Initial GCS score	0.901 (0.824-0.985)	0.0217*
Initial NIHSS	1.109 (1.043-1.179)	0.0028**
NIHSS 24 hours after ER arrival	1.158 (1.073-1.249)	0.0002**
SBP on ER arrival	1 (0.993-1.008)	0.9214
SBP 24 hours after ER arrival	1.009 (0.992-1.027)	0.3061
SBP on first rehabilitation	1 (0.984-1.017)	0.9866
Surgical management	1.556 (0.836-2.896)	0.1634
Hematoma location		
Lobar	1.266 (0.714-2.243)	0.4199
Putamen	0.448 (0.232-0.862)	0.0165*
Caudate nucleus	0.219 (0.011-4.458)	0.3236
Thalamus	1.603 (0.901-2.852)	0.1087
Brainstem	1.107 (0.39-3.143)	0.8492
Cerebellum	0.643 (0.236-1.748)	0.3868
ICH volume	1.014 (1.003-1.025)	0.0106*
Intraventricular hemorrhage	2.584 (1.359-4.912)	0.0038**
ICH score	1.756 (1.308-2.358)	0.0002**
**Activity-based indicators**		
Onset-to-first PT time	1.01 (1-1.019)	0.0418*
Initial ability to sit independently without physical assistance	0.522 (0.403-0.676)	<0.0001**
Initial ability to sit independently for 2 minutes	0.533 (0.414-0.687)	<0.0001**
Immobility adverse events	2.205 (1.242-3.916)	0.0069**

Total hospitalization days	1.024 (1.011-1.037)	0.0002**

Abbreviation: BMI, body mass index; mRS, modified Rankin Scale; Hb, hemoglobin; WBC, white blood cell; CRP, C-reactive protein; eGFR, estimated glomerular filtration rate; GCS, Glasgow Coma Scale; NIHSS, National Institute of Health Stroke Scale; SBP, systolic blood pressure; DBP, diastolic blood pressure; ER: emergency room; PT, physical therapy; ICH, intracerebral hemorrhage; OR, odds ratio; CI, confidence interval. * p < 0.05; **p < 0.01

S-Table 2 Association between initial sitting ability and the "transfer" item in the Barthel index at 4 weeks

**Parameters**	**"Transfer" item in Barthel index at 4 weeks**	
	OR (95% CI)	p value
**Initial ability to sit independently without physical assistance**		
Dependent (vs. independent)	2.046 (1.073, 3.902)	0.0297 *
**Initial ability to sit independently for 2 minutes**		
Dependent (vs. independent)	4.016 (2.320, 6.944)	<0.0001 **

Abbreviation: OR, odds ratio; CI, confidence interval. * p < 0.05; **p < 0.01

S-Table 3a Univariate analysis: Factors associated with Brunnstrom stage of hemiplegic proximal upper limb at 4 weeks

**Parameter**	**Unadjusted OR (95% CI)**	**p value**
**Baseline status**		
Age	0.993 (0.987-0.999)	0.0256*
Male	1.055 (0.901-1.235)	0.5061
BMI	1.011 (0.993-1.029)	0.2208
Premorbid mRS	0.968 (0.887-1.057)	0.4695
**Risk factors**		
Hypertension	1.107 (0.928-1.320)	0.2585
Diabetes mellitus	0.886 (0.737-1.065)	0.1977
Dyslipidemia	0.948 (0.795-1.131)	0.5557
Atrial fibrillation	0.895 (0.618-1.296)	0.5569
Peripheral Arterial Occlusion Disease	0.559 (0.165-1.897)	0.3508
Ischemic heart disease	0.901 (0.679-1.196)	0.4704
Previous history of ischemic stroke	0.718 (0.543-0.950)	0.0204*

Previous history of hemorrhagic stroke	1.092 (0.862-1.384)	0.4643
Smoking	1.016 (0.818-1.261)	0.8871
**Initial laboratory data**		
Hb	1.012 (0.975-1.050)	0.5258
Platelet count	1.001 (1.000-1.002)	0.0208*
WBC count	1.005 (0.981-1.030)	0.6674
Blood glucose	1.001 (1.000-1.002)	0.1878
CRP	0.993 (0.976-1.011)	0.4496
Albumin	0.929 (0.768-1.124)	0.4484
Total cholesterol	1.001 (0.999-1.004)	0.1948
eGFR	1.002 (1.000-1.004)	0.1341
**Previous medication use**		
Antiplatelet drugs	0.888 (0.646-1.220)	0.4631
Anticoagulant drugs	0.870 (0.678-1.117)	0.2754
Antihypertensive drugs	1.004 (0.856-1.178)	0.9602
Lipid-lowering drugs	1.028 (0.830-1.273)	0.7988
**ICH-related characteristics**		
Initial GCS score	0.961 (0.937-0.985)	0.0020**
Initial NIHSS	1.062 (1.041-1.084)	<.0001**
NIHSS 24 hours after ER arrival	1.090 (1.061-1.119)	<.0001**
SBP on ER arrival	1.001 (0.998-1.003)	0.6399
SBP 24 hours after ER arrival	1.003 (0.998-1.007)	0.2880
SBP on first rehabilitation	0.998 (0.993-1.003)	0.3877
Surgical management	1.325 (1.104-1.590)	0.0025**
Hematoma location		
Lobar	1.028 (0.849-1.244)	0.7779
Putamen	1.171 (0.989-1.386)	0.0666
Caudate nucleus	0.310 (0.046-2.084)	0.2282
Thalamus	1.011 (0.859-1.191)	0.8914

Brainstem	0.825 (0.600-1.135)	0.2365
Cerebellum	0.381 (0.223-0.651)	0.0004**
ICH volume	1.011 (1.007-1.015)	<.0001**
Intraventricular hemorrhage	1.113 (0.948-1.308)	0.1908
ICH score	1.138 (1.050-1.234)	0.0017**
**Activity-based indicators**		
Onset-to-first PT time	1.002 (0.999-1.006)	0.1566
Initial ability to sit independently without physical assistance	0.692 (0.626-0.766)	<.0001**
Initial ability to sit independently for 2 minutes	0.727 (0.665-0.795)	<.0001**
Immobility adverse events	1.285 (1.092-1.511)	0.0025**
Total hospitalization days	1.007 (1.004-1.011)	<.0001**

Abbreviation: BMI, body mass index; mRS, modified Rankin Scale; Hb, hemoglobin; WBC, white blood cell; CRP, C-reactive protein; eGFR, estimated glomerular filtration rate; GCS, Glasgow Coma Scale; NIHSS, National Institute of Health Stroke Scale; SBP, systolic blood pressure; DBP, diastolic blood pressure; ER: emergency room; PT, physical therapy; ICH, intracerebral hemorrhage; OR, odds ratio; CI, confidence interval. * $p < 0.05$; ** $p < 0.01$

S-Table 3b Univariate analysis: Factors associated with Brunnstrom stage of hemiplegic distal upper limb at 4 weeks

Parameter	Unadjusted OR (95% CI)	p value
**Baseline status**		
Age	0.993 (0.988-0.999)	0.0198*
Male	1.059 (0.918-1.220)	0.4317
BMI	1.010 (0.994-1.026)	0.2313
Premorbid mRS	0.949 (0.874-1.029)	0.2069
**Risk factors**		
Hypertension	1.053 (0.899-1.233)	0.5225
Diabetes mellitus	0.875 (0.741-1.034)	0.1174
Dyslipidemia	0.959 (0.818-1.124)	0.6013
Atrial fibrillation	0.962 (0.694-1.335)	0.8186
Peripheral Arterial Occlusion Disease	0.576 (0.186-1.787)	0.3398

Ischemic heart disease	0.921 (0.715-1.187)	0.5267
Previous history of ischemic stroke	0.685 (0.526-0.891)	0.0049**
Previous history of hemorrhagic stroke	1.033 (0.833-1.280)	0.7690
Smoking	0.986 (0.810-1.200)	0.8879
**Initial laboratory data**		
Hb	0.996 (0.964-1.030)	0.8284
Platelet count	1.001 (1.000-1.002)	0.0228*
WBC count	1.008 (0.986-1.031)	0.4557
Blood glucose	1.000 (0.999-1.002)	0.5021
CRP	0.995 (0.980-1.010)	0.5172
Albumin	0.907 (0.764-1.076)	0.2647
Total cholesterol	1.001 (0.999-1.003)	0.3501
eGFR	1.001 (0.999-1.003)	0.1710
**Previous medication use**		
Antiplatelet drugs	0.927 (0.699-1.230)	0.5994
Anticoagulant drugs	0.845 (0.673-1.062)	0.1486
Antihypertensive drugs	0.946 (0.818-1.093)	0.4502
Lipid-lowering drugs	1.046 (0.862-1.269)	0.6491
**ICH-related characteristics**		
Initial GCS score	0.967 (0.945-0.990)	0.0046**
Initial NIHSS	1.059 (1.039-1.079)	<.0001**
NIHSS 24 hours after ER arrival	1.086 (1.059-1.114)	<.0001**
SBP on ER arrival	1.001 (0.998-1.003)	0.6177
SBP 24 hours after ER arrival	1.003 (0.998-1.007)	0.2313
SBP on first rehabilitation	1.000 (0.995-1.004)	0.9234
Surgical management	1.293 (1.096-1.526)	0.0023**
Hematoma location		
Lobar	1.053 (0.886-1.252)	0.5554
Putamen	1.205 (1.034-1.406)	0.0173*

Caudate nucleus	0.344 (0.057-2.088)	0.2462
Thalamus	0.953 (0.822-1.106)	0.5256
Brainstem	0.853 (0.642-1.134)	0.2736
Cerebellum	0.414 (0.252-0.680)	0.0005**
ICH volume	1.012 (1.008-1.016)	<.0001**
Intraventricular hemorrhage	1.096 (0.948-1.268)	0.2151
ICH score	1.121 (1.042-1.206)	0.0021**
**Activity-based indicators**		
Onset-to-first PT time	1.002 (0.999-1.005)	0.2021
Initial ability to sit independently without physical assistance	0.736 (0.675-0.803)	<.0001**
Initial ability to sit independently for 2 minutes	0.766 (0.709-0.828)	<.0001**
Immobility adverse events	1.212 (1.048-1.403)	0.0097**
Total hospitalization days	1.007 (1.003-1.010)	0.0001**

Abbreviation: BMI, body mass index; mRS, modified Rankin Scale; Hb, hemoglobin; WBC, white blood cell; CRP, C-reactive protein; eGFR, estimated glomerular filtration rate; GCS, Glasgow Coma Scale; NIHSS, National Institute of Health Stroke Scale; SBP, systolic blood pressure; DBP, diastolic blood pressure; ER: emergency room; PT, physical therapy; ICH, intracerebral hemorrhage; OR, odds ratio; CI, confidence interval. * $p < 0.05$; ** $p < 0.01$

S-Table 4a Univariate analysis: Factors associated with Brunnstrom stage of hemiplegic proximal upper limb at 12 weeks

Parameter	Unadjusted OR (95% CI)	p value
**Baseline status**		
Age	0.992 (0.982-1.002)	0.1188
Male	0.994 (0.763-1.284)	0.9642
BMI	1.013 (0.985-1.042)	0.3614
Premorbid mRS	0.897 (0.748-1.077)	0.2440
**Risk factors**		
Hypertension	0.979 (0.732-1.304)	0.8830
Diabetes mellitus	0.890 (0.662-1.195)	0.4375
Dyslipidemia	1.042 (0.788-1.378)	0.7740
Atrial fibrillation	0.797 (0.433-1.467)	0.4666

Peripheral Arterial Occlusion Disease	0.806 (0.149-4.348)	0.8020
Ischemic heart disease	0.736 (0.424-1.277)	0.2751
Previous history of ischemic stroke	0.898 (0.627-1.287)	0.5577
Previous history of hemorrhagic stroke	0.987 (0.663-1.467)	0.9465
Smoking	0.842 (0.576-1.229)	0.3718
**Initial laboratory data**		
Hb	0.986 (0.922-1.055)	0.6905
Platelet count	1.000 (0.998-1.002)	0.7534
WBC count	0.992 (0.955-1.031)	0.6927
Blood glucose	1.002 (1.000-1.004)	0.1188
CRP	0.997 (0.976-1.019)	0.8001
Albumin	0.749 (0.535-1.048)	0.0916
Total cholesterol	1.002 (0.998-1.006)	0.3327
eGFR	0.998 (0.994-1.001)	0.2205
**Previous medication use**		
Antiplatelet drugs	0.792 (0.478-1.312)	0.3651
Anticoagulant drugs	0.850 (0.574-1.260)	0.4196
Antihypertensive drugs	0.930 (0.712-1.213)	0.5916
Lipid-lowering drugs	1.021 (0.724-1.439)	0.9065
**ICH-related characteristics**		
Initial GCS score	0.943 (0.906-0.980)	0.0032**
Initial NIHSS	1.075 (1.04-1.1112)	<0.0001**
NIHSS 24 hours after ER arrival	1.107 (1.059-1.158)	<0.0001**
SBP on ER arrival	1.003 (0.999-1.008)	0.1170
SBP 24 hours after ER arrival	1.001 (0.993-1.009)	0.8467
SBP on first rehabilitation	1.000 (0.991-1.009)	0.9681
Surgical management	1.290 (0.964-1.725)	0.0865
Hematoma location		
Lobar	1.052 (0.749-1.479)	0.7691

Putamen	1.071 (0.810-1.418)	0.6298
Caudate nucleus	0.472 (0.079-2.797)	0.4079
Thalamus	1.091 (0.836-1.424)	0.5221
Brainstem	0.935 (0.599-1.460)	0.7688
Cerebellum	0.484 (0.261-0.899)	0.0215*
ICH volume	1.015 (1.009-1.022)	<0.0001**
Intraventricular hemorrhage	1.076 (0.826-1.402)	0.5868
ICH score	1.227 (1.074-1.401)	0.0025**
**Activity-based indicators**		
Onset-to-first PT time	1.004 (0.997-1.011)	0.2355
Initial ability to sit independently without physical assistance	0.610 (0.506-0.735)	<0.0001**
Initial ability to sit independently for 2 minutes	0.705 (0.611-0.812)	<0.0001**
Immobility adverse events	1.340 (1.026-1.749)	0.0315*
Hospitalization days	1.015 (1.009-1.022)	<0.0001**

Abbreviation: BMI, body mass index; mRS, modified Rankin Scale; Hb, hemoglobin; WBC, white blood cell; CRP, C-reactive protein; eGFR, estimated glomerular filtration rate; GCS, Glasgow Coma Scale; NIHSS, National Institute of Health Stroke Scale; SBP, systolic blood pressure; DBP, diastolic blood pressure; ER: emergency room; PT, physical therapy; ICH, intracerebral hemorrhage; OR, odds ratio; CI, confidence interval. * $p < 0.05$; ** $p < 0.01$

S-Table 4b Univariate analysis: Factors associated with Brunnstrom stage of hemiplegic distal upper limb at 12 weeks

Parameter	Unadjusted OR (95% CI)	p value
**Baseline status**		
Age	0.991 (0.982-1.000)	0.0600
Male	1.061 (0.832-1.355)	0.6322
BMI	1.022 (0.997-1.050)	0.0871
Premorbid mRS	0.903 (0.762-1.070)	0.2378
**Risk factors**		
Hypertension	0.910 (0.704-1.177)	0.4731
Diabetes mellitus	0.867 (0.658-1.143)	0.3114
Dyslipidemia	1.045 (0.810-1.348)	0.7357

Atrial fibrillation	0.922 (0.552-1.540)	0.7559
Peripheral Arterial Occlusion Disease	0.801 (0.164-3.903)	0.7831
Ischemic heart disease	0.737 (0.438-1.240)	0.2499
Previous history of ischemic stroke	0.900 (0.648-1.251)	0.5312
Previous history of hemorrhagic stroke	1.050 (0.740-1.490)	0.7840
Smoking	0.831 (0.580-1.191)	0.314
**Initial laboratory data**		
Hb	0.993 (0.934-1.056)	0.8249
Platelet count	1.000 (0.998-1.002)	0.9872
WBC count	0.991 (0.957-1.026)	0.6213
Blood glucose	1.002 (0.999-1.004)	0.1390
CRP	0.995 (0.973-1.016)	0.6245
Albumin	0.770 (0.562-1.055)	0.1042
Total cholesterol	1.001 (0.998-1.005)	0.4495
eGFR	0.998 (0.995-1.001)	0.1883
**Previous medication use**		
Antiplatelet drugs	0.832 (0.528-1.310)	0.4267
Anticoagulant drugs	0.835 (0.579-1.206)	0.3365
Antihypertensive drugs	0.946 (0.743-1.204)	0.6513
Lipid-lowering drugs	1.027 (0.754-1.400)	0.8646
**ICH-related characteristics**		
Initial GCS score	0.952 (0.919-0.986)	0.0062**
Initial NIHSS	1.064 (1.032-1.096)	<0.0001**
NIHSS 24 hours after ER arrival	1.077 (1.039-1.116)	<0.0001**
SBP on ER arrival	1.001 (0.998-1.005)	0.4826
SBP 24 hours after ER arrival	1.002 (0.994-1.010)	0.6158
SBP on first rehabilitation	1.000 (0.992-1.007)	0.9059
Surgical management	1.261 (0968-1.643)	0.0853
Hematoma location		

Lobar	1.031 (0.737-1.391)	0.9386
Putamen	1.043 (0.805-1.351)	0.7504
Caudate nucleus	0.488 (0.085-2.802)	0.4212
Thalamus	1.069 (0.839-1.362)	0.5901
Brainstem	0.989 (0.667-1.466)	0.9557
Cerebellum	0.502 (0.275-0.916)	0.0248*
ICH volume	1.015 (1.009-1.021)	<0.0001**
Intraventricular hemorrhage	1.062 (0.834-1.351)	0.6271
ICH score	1.219 (1.078-1.378)	0.0016**
**Activity-based indicators**		
Onset-to-first PT time	1.004 (0.998-1.010)	0.1663
Initial ability to sit independently without physical assistance	0.627 (0.524-0.750)	<0.0001**
Initial ability to sit independently for 2 minutes	0.722 (0.631-0.825)	<0.0001**
Immobility adverse events	1.184 (0.935-1.501)	0.1615
Total hospitalization days	1.013 (1.007-1.018)	<0.0001**

Abbreviation: BMI, body mass index; mRS, modified Rankin Scale; Hb, hemoglobin; WBC, white blood cell; CRP, C-reactive protein; eGFR, estimated glomerular filtration rate; GCS, Glasgow Coma Scale; NIHSS, National Institute of Health Stroke Scale; SBP, systolic blood pressure; DBP, diastolic blood pressure; ER: emergency room; PT, physical therapy; ICH, intracerebral hemorrhage; OR, odds ratio; CI, confidence interval. * $p < 0.05$; ** $p < 0.01$

S-Table 5a Univariate analysis: Factors associated with Brunnstrom stage of hemiplegic lower limb at 4 weeks

Parameter	Unadjusted OR (95% CI)	p value
**Baseline status**		
Age	0.992 (0.985-1.000)	0.0369*
Male	1.079 (0.896-1.298)	0.4229
BMI	1.011 (0.990-1.031)	0.3175
Premorbid mRS	1.010 (0.914-1.116)	0.8436
**Risk factors**		
Hypertension	1.115 (0.906-1.372)	0.3048
Diabetes mellitus	0.972 (0.786-1.202)	0.7926

Dyslipidemia	0.997 (0.812-1.225)	0.9770
Atrial fibrillation	0.978 (0.639-1.495)	0.9164
Peripheral Arterial Occlusion Disease	0.564 (0.148-2.158)	0.4027
Ischemic heart disease	0.879 (0.629-1.230)	0.4526
Previous history of ischemic stroke	0.722 (0.552-0.999)	0.0490*
Previous history of hemorrhagic stroke	1.200 (0.912-1.579)	0.1919
Smoking	1.082 (0.841-1.392)	0.5390
**Initial laboratory data**		
Hb	1.019 (0.975-1.064)	0.4090
Platelet count	1.001 (1.000-1.002)	0.1039
WBC count	1.008 (0.980-1.037)	0.5887
Blood glucose	1.001 (1.000-1.003)	0.0894
CRP	0.996 (0.977-1.016)	0.6981
Albumin	0.850 (0.680-1.062)	0.1522
Total cholesterol	1.001 (0.999-1.004)	0.2722
eGFR	1.001 (0.998-1.003)	0.5006
**Previous medication use**		
Antiplatelet drugs	0.852 (0.583-1.244)	0.4068
Anticoagulant drugs	0.820 (0.608-1.107)	0.1947
Antihypertensive drugs	0.951 (0.788-1.148)	0.6013
Lipid-lowering drugs	1.064 (0.829-1.366)	0.6253
**ICH-related characteristics**		
Initial GCS score	0.945 (0.917-0.974)	0.0002**
Initial NIHSS	1.077 (1.050-1.104)	<0.0001**
NIHSS 24 hours after ER arrival	1.113 (1.076-1.150)	<0.0001**
SBP on ER arrival	1.000 (0.997-1.003)	0.8083
SBP 24 hours after ER arrival	1.000 (0.995-1.006)	0.8646
SBP on first rehabilitation	0.997 (0.992-1.003)	0.3980
Surgical management	1.455 (1.175-1.802)	0.0006**

Hematoma location		
Lobar	1.049 (0.839-1.311)	0.6773
Putamen	1.113 (0.915-1.355)	0.2837
Caudate nucleus	0.291 (0.042-2.019)	0.2117
Thalamus	1.043 (0.861-1.263)	0.6667
Brainstem	0.804 (0.551-1.173)	0.2567
Cerebellum	0.333 (0.184-0.602)	0.0003**
ICH volume	1.013 (1.009-1.018)	<0.0001**
Intraventricular hemorrhage	1.258 (1.040-1.522)	0.0182*
ICH score	1.223 (1.109-1.349)	<0.0001**
**Activity-based indicators**		
Onset-to-first PT time	1.003 (0.999-1.007)	0.1398
Initial ability to sit independently without physical assistance	0.606 (0.532-0.691)	<0.0001**
Initial ability to sit independently for 2 minutes	0.646 (0.575-0.724)	<0.0001**
Immobility adverse events	1.414 (1.163-1.719)	0.0005**
Total hospitalization days	1.009 (1.004-1.013)	<0.0001**

Abbreviation: BMI, body mass index; mRS, modified Rankin Scale; Hb, hemoglobin; WBC, white blood cell; CRP, C-reactive protein; eGFR, estimated glomerular filtration rate; GCS, Glasgow Coma Scale; NIHSS, National Institute of Health Stroke Scale; SBP, systolic blood pressure; DBP, diastolic blood pressure; ER: emergency room; PT, physical therapy; ICH, intracerebral hemorrhage; OR, odds ratio; CI, confidence interval. * $p < 0.05$; ** $p < 0.01$

S-Table 5b Univariate analysis: Factors associated with Brunnstrom stage of hemiplegic lower limb at 12 weeks

Parameter	Unadjusted OR (95% CI)	p value
**Baseline status**		
Age	0.990 (0.977-1.002)	0.1093
Male	1.027 (0.733-1.438)	0.8780
BMI	1.017 (0.981-1.054)	0.3491
Premorbid mRS	0.942 (0.765-1.159)	0.5713
**Risk factors**		
Hypertension	1.048 (0.725-1.514)	0.8043

Diabetes mellitus	0.856 (0.587-1.250)	0.4212
Dyslipidemia	0.981 (0.684-1.406)	0.9167
Atrial fibrillation	1.005 (0.508-1.985)	0.9896
Peripheral Arterial Occlusion Disease	0.891 (0.125-6.362)	0.9084
Ischemic heart disease	0.548 (0.257-1.167)	0.1187
Previous history of ischemic stroke	0.968 (0.623-1.504)	0.8855
Previous history of hemorrhagic stroke	1.138 (0.703-1.844)	0.5983
Smoking	0.824 (0.509-1.335)	0.4321
**Initial laboratory data**		
Hb	1.002 (0.920-1.092)	0.9617
Platelet count	1.000 (0.997-1.002)	0.7735
WBC count	0.994 (0.948-1.044)	0.8203
Blood glucose	1.002 (0.999-1.005)	0.1656
CRP	0.989 (0.959-1.021)	0.5060
Albumin	0.765 (0.494-1.186)	0.2318
Total cholesterol	1.003 (0.997-1.008)	0.3221
eGFR	0.998 (0.993-1.002)	0.2994
**Previous medication use**		
Antiplatelet drugs	0.573 (0.282-1.165)	0.1240
Anticoagulant drugs	0.670 (0.392-1.144)	0.1424
Antihypertensive drugs	0.912 (0.651-1.278)	0.5941
Lipid-lowering drugs	0.968 (0.623-1.504)	0.8855
**ICH-related characteristics**		
Initial GCS score	0.910 (0.863-0.959)	0.0004**
Initial NIHSS	1.088 (1.044-1.133)	<.0001**
NIHSS 24 hours after ER arrival	1.136 (1.072-1.204)	<.0001**
SBP on ER arrival	1.004 (0.999-1.010)	0.1181
SBP 24 hours after ER arrival	1.000 (0.990-1.011)	0.9540
SBP on first rehabilitation	0.996 (0.985-1.007)	0.5177

Surgical management	1.406 (0.967-2.042)	0.0740
Hematoma location		
Lobar	1.077 (0.696-1.667)	0.7382
Putamen	0.955 (0.660-1.380)	0.8043
Caudate nucleus	0.452 (0.066-3.087)	0.4176
Thalamus	1.109 (0.791-1.556)	0.5471
Brainstem	0.881 (0.498-1.558)	0.6635
Cerebellum	0.412 (0.200-0.848)	0.0160*
ICH volume	1.017 (1.009-1.025)	<.0001**
Intraventricular hemorrhage	1.104 (0.789-1.547)	0.5630
ICH score	1.340 (1.125-1.597)	0.0010**
**Activity-based indicators**		
Onset-to-first PT time	1.006 (0.998-1.014)	0.1614
Initial ability to sit independently without physical assistance	0.542 (0.433-0.679)	<.0001**
Initial ability to sit independently for 2 minutes	0.609 (0.506-0.733)	<.0001**
Immobility adverse events	1.364 (0.974-1.908)	0.0705
Total hospitalization days	1.021 (1.012-1.030)	<.0001**

Abbreviation: BMI, body mass index; mRS, modified Rankin Scale; Hb, hemoglobin; WBC, white blood cell; CRP, C-reactive protein; eGFR, estimated glomerular filtration rate; GCS, Glasgow Coma Scale; NIHSS, National Institute of Health Stroke Scale; SBP, systolic blood pressure; DBP, diastolic blood pressure; ER: emergency room; PT, physical therapy; ICH, intracerebral hemorrhage; OR, odds ratio; CI, confidence interval. * $p < 0.05$; ** $p < 0.01$

S-Table 6a Association between medication use post-ICH during acute care and post-ICH immobility adverse events

Medication use post-ICH during acute care	Immobility adverse events		p value
	Yes	No	
Mannitol	40 (27.03)	37 (23.87)	0.5282
Corticosteroids	34 (22.97)	17 (10.97)	0.0052**
Benzodiazepines	83 (56.08)	62 (40.00)	0.0051**
Opioids	24 (16.22)	27 (17.42)	0.7796

Abbreviation: ICH, intracerebral hemorrhage. * $p < 0.05$; ** $p < 0.01$

S-Table 6b Association between medication use post-ICH during acute care and initial sitting ability

Medication use post-ICH during acute care	Initial ability to sit without physical assistance			
	Dependent (N=294)	Independent (N=12)	OR (95% CI)	p value
	N (%)	N (%)		
Mannitol	77 (26.19%)	2 (16.67%)	1.774 (0.380,8.278)	0.4657
Corticosteroids	50 (17.01%)	1 (8.33%)	2.254 (0.285,17.852)	0.4415
Benzodiazepines	145 (49.32%)	3 (25.00%)	2.919 (0.775,10.998)	0.1134
Opioids	242 (82.31%)	8 (66.67%)	0.430 (0.125,1.480)	0.1808
**Medication use post-ICH during acute care**	**Initial ability to sit independently for 2 minutes**			
	Dependent (N=233)	Independent (N=73)	OR (95% CI)	p value
	N (%)	N (%)		
Mannitol	57 (24.46%)	22 (30.14%)	0.751 (0.419,1.344)	0.3346
Corticosteroids	33 (14.16%)	18 (24.66%)	0.504 (0.264,0.963)	0.0381*
Benzodiazepines	111 (47.64%)	37 (50.68%)	0.885 (0.523,1.498)	0.6497

Opioids	195 (83.69%)	55 (75.34%)	0.595 (0.315,1.124)	0.1097

Abbreviation: ICH, intracerebral hemorrhage; OR, odds ratio; CI, confidence interval. * p < 0.05; **p < 0.01

S-Table 7a Association between surgery and length of hospital stay

	N	Total hospitalization days (mean ± SD)	p value
Non-surgical group	232	40.35 ± 23.56	0.1029
Surgical group	78	45.40 ± 23.20	

Abbreviation: SD, standard deviation

S-Table 7b Association between length of hospital stay and Brunnstrom stage of hemiplegic limb in surgical vs non-surgical groups

**Non-surgical group**				
**Parameters**	**Upper proximal limb at 4 weeks**		**Upper proximal limb at 12 weeks**	
	OR (95% CI)	p value	OR (95% CI)	p value
Total hospitalization days	1.009 (1.004,1.014)	0.0001**	1.018 (1.010,1.027)	<0.0001 **
**Parameters**	**Upper distal limb at 4 weeks**		**Upper distal limb at 12 weeks**	
	OR (95% CI)	p value	OR (95% CI)	p value
Total hospitalization days	1.008 (1.004,1.012)	0.0002 **	1.015 (1.008,1.022)	<0.0001 **
**Parameters**	**Lower limb at 4 weeks**		**Lower limb at 12 weeks**	
	OR (95% CI)	p value	OR (95% CI)	p value
Total hospitalization days	1.011 (1.005, 1.016)	0.0001**	1.024 (1.013,1.036)	<0.0001 **
**Surgical group**				

Parameters	Upper proximal limb at 4 weeks		Upper proximal limb at 12 weeks	
	OR (95% CI)	p value	OR (95% CI)	p value
Total hospitalization days	1.003 (0.996,1.009)	0.4114	1.010 (1.000,1.021)	0.0501
**Parameters**	**Upper distal limb at 4 weeks**		**Upper distal limb at 12 weeks**	
	OR (95% CI)	p value	OR (95% CI)	p value
Total hospitalization days	1.003 (0.996,1.009)	0.3830	1.008 (0.999,1.018)	0.0684
	**Lower limb at 4 weeks**		**Lower limb at 12 weeks**	
**Parameters**	OR (95% CI)	p value	OR (95% CI)	p value
Total hospitalization days	1.004 (0.997,1.011)	0.3017	1.016 (1.002,1.029)	0.0249 *

Abbreviation: OR, odds ratio; CI, confidence interval. * $p < 0.05$; ** $p < 0.01$
